# Exploring Factors Influencing Caregiver Burden: A Systematic Review of Family Caregivers of Older Adults with Chronic Illness in Local Communities

**DOI:** 10.3390/healthcare12101002

**Published:** 2024-05-13

**Authors:** Jin Young Choi, Seon Heui Lee, Soyoung Yu

**Affiliations:** 1 Graduate School of Nursing, Gachon University, Incheon 21936, Republic of Korea; naokko33@gilhospital.com; 2 Department of Nursing Science, College of Nursing, Gachon University, 191 Hambakmoero, Yeonsu-gu, Incheon 21936, Republic of Korea; 3 College of Nursing, CHA University, 120, Pocheon 11160, Republic of Korea

**Keywords:** older adult, chronic illness, caregiver burden, systematic review, tailored interventions

## Abstract

This study aimed to systematically review and analyze factors contributing to caregiver burden among family caregivers of older adults with chronic illnesses in local communities. Specific objectives included exploring the characteristics of older adults with chronic illness and caregiver burden through an extensive literature review and identifying factors influencing caregiver burden in this population. Using Korean (RISS, KISS, and KoreaMed) and international (EMBASE, MEDLINE, CINAHL, and the Cochrane Library) databases, this study employed systematic search methods to identify relevant literature. The inclusion and exclusion criteria were systematically applied in accordance with the PRISMA guidelines, focusing on studies that addressed caregiver burden among family caregivers of older adults with chronic illnesses in local communities. Following the database search, 15,962 articles were identified. After eliminating duplicates and applying the selection criteria, 18 studies were included in this review. These studies, representing various countries, contribute to a diverse dataset covering caregiver and care-recipient characteristics, including age, sex, chronic conditions, and various caregiver burden assessment tools. This systematic review provides a comprehensive understanding of the factors that influence caregiver burden among family caregivers of older adults with chronic illness in local communities. These findings emphasize the need for integrated nursing interventions and community efforts to address the welfare concerns of this population and support their caregivers.

## 1. Introduction

The proportion of the population aged 65 or over is increasing worldwide. By 2050, more than two billion of the world’s population will be aged 65 years or older. The Elderly Welfare Act stipulates that individuals aged 65 years and older should be considered older adults, whereas the National Basic Living Security Act adopts the same age threshold as defined in the Elderly Welfare Act. The aged population is defined as individuals aged 65 years or older, according to the OECD [1]. Similarly, the World Report on Aging and Health defines older adults as individuals aged 65 years or older. In Europe, the most rapidly aging region, this proportion will exceed 30% [1]. The average life expectancy of humans has increased worldwide, which has led to rapid aging. In Korea, 18.4% of the population was aged 65 years or older in 2023; this proportion is expected to exceed 20.6% by 2025, entering a super-aged society, and 30% by 2035 [2]. With the increase in the older adult population, many older people suffer from chronic diseases and difficulties in life owing to physical, mental, and social deterioration. Older people experience certain health problems, such as cancer, hip fracture, stroke, and dementia, at higher rates than younger people and are more likely to have comorbid conditions that lead to higher care and support needs [3]. Comorbidity refers to the simultaneous presence of two or more medical conditions in a patient. Individuals with comorbidities demonstrate higher rates of hospital admission, prolonged hospitalization, and increased utilization of long-term care services. Consequently, this contributes to elevated medical expenses and imposes a substantial economic burden [4]. In Korea, 74.3% of all deaths are due to chronic diseases, and the prevalence of major chronic diseases is increasing. Cancer, heart disease, cerebrovascular disease, diabetes, and chronic lower respiratory disease are the top five leading causes of death in Korea, indicating that decline in function due to aging and active management of chronic diseases are becoming critical issues [5].

The global phenomenon of an aging population has the dual effect of increasing the number of older people with physical and mental disabilities requiring care and the number of caregivers required for older people with disabilities [6]. As the population ages, the number of chronically ill older people living in communities increases, leading to prolonged caregiving, resulting in sleeplessness, fatigue, and emotional instability among family caregivers [7].

Home care has been recognized as having a stressful impact on caregivers’ health and quality of life. Caregivers often feel unprepared for their new roles, which can lead to distress and the deterioration of their physical, mental, and social health. These negative effects have been described in terms of burden, strain, and stress. Informal care is defined as the provision of unpaid assistance to individuals with varying degrees of dependency and is typically administered by family members. This mode of care constitutes 80–90% of dependency support and is predominantly administered within the home setting. The number of informal caregivers across various European nations ranges from 20% to 44% of the total population. In the conventional care paradigm, women predominantly undertake the bulk of unpaid caregiving responsibilities, comprising approximately 80% of informal caregivers globally, with comparable figures observed in Europe. Notably, women aged 45–60 have emerged as primary providers of informal care across all European countries [8]. Caregiver burden refers to the negative emotions and strain experienced by caregivers as a result of caring for patients with chronic illnesses. It is a negative outcome of the caregiving experience, exacerbated by the multiple roles and responsibilities that caregivers fulfill [9]. Consequently, numerous studies have been conducted on the caregiving burden of community-dwelling older adults with chronic diseases. In addition, interventions for older adults with chronic diseases and their families are being conducted. Community services, such as community-based chronic disease management initiatives, are being deployed to alleviate the caregiving burden among caregivers of individuals with chronic disease. These programs also aim to address chronic disease management on a national scale. To identify the various negative effects of caregiving, this study examines the concepts of depression, stress, caregiver burden, and exhaustion [6,7,9,10].

Previous research on caregiver burden and stressors has identified demographic, clinical, and psychological characteristics as factors that influence caregiver stress [6,7,9,10]. In terms of demographic characteristics, (1) female caregivers were more likely to have higher stress, (2) longer duration of illness was associated with higher caregiver stress, (3) dementia and stroke were the most common types of illness associated with caregiver stress, and (4) higher caregiver-related demographic stress was associated with higher caregiver stress. Meanwhile, spouses, sons, and daughters-in-law were more likely to have higher caregiver stress in terms of their relationship with the patient. In terms of clinical-related characteristics, (1) caregivers experienced more stress as functional status declined and cognitive decline increased; (2) caregivers of individuals with neurobehavioral symptoms experienced more stress; and (3) caregivers’ psychological characteristics, such as distress, depression, and social support, contributed to caregiver stress. Caregiver burden was found to be negatively correlated with positive concepts, such as caregiver social support, and positively correlated with negative concepts, such as depression and distress [11]. Furthermore, caregiver burden was higher when patient depression was severe; caregiver pain was greater because of the patient’s neuropsychiatric behavioral symptoms, and caregiver depression was severe. Psychiatric behavioral symptoms encompass prevalent non-cognitive functional neuropsychiatric manifestations subsequent to stroke, including depressive disorders, anxiety disorders, post-traumatic stress disorders, and psychotic disorders [12]. Previous research on caregiving has focused on caregiving issues, with a particular focus on dementia. In addition, several studies have been conducted on the caregiving burden of older adults living in institutions such as nursing homes and daycare centers or using social services. However, research that systematically identifies the caregiving burden on family caregivers of community-dwelling older adults with chronic diseases is lacking. Therefore, this study’s primary aim was to meticulously examine and systematically assess the factors that influence caregiving burden experienced by caregivers tasked with managing chronic illnesses in their communities. The goal was to identify the specific determinants contributing to this burden, with the overarching objective of establishing a foundation for the development of interventions aimed at alleviating caregiver stress.

### Purpose of the Present Study

This study aimed to systematically review factors related to family caregiver burden in community-dwelling older adults with chronic diseases and calculate the effect size of each factor to verify statistical significance. The objectives of this study were as follows:

(1) To identify characteristics of chronically ill community-dwelling older adults and their family caregivers’ burden through a systematic review.
(2) To identify factors affecting caregiving burden of family caregivers of community-dwelling older adults with chronic diseases through a systematic review.

## 2. Methods

### 2.1. Research Design

This study was a systematic review of family caregiver burden among community-dwelling chronically ill older adults to identify and analyze factors associated with caregiver burden.

### 2.2. Data Sources

This study employed a systematic review method to search the literature using RISS, KISS, KoreaMed, EMBASE, MEDLINE (Ovid-MEDLINE), CINAHL, and the Cochrane Library to identify studies on caregiving burden factors for family caregivers of community-dwelling chronically ill older adults. The search terms were set as follows: (i) Community OR communities OR community care OR Long-term care OR community-dwelling OR home-dwelling OR community-based, (ii) aged OR old OR older OR elderly OR elderly people OR elderly person OR older people OR elderly population OR older person OR older patients OR older adults OR aging population OR aging OR silver OR elderly human OR impaired elderly OR disability OR disabled elderly OR frail elderly OR frail elderly person, (iii) caregiver OR caregivers OR spouse OR spousal caregiver OR carers OR care providers OR caregiving OR family carers OR family caregiver OR family caregivers OR informal carers OR informal caregiver OR informal caregivers OR primary caregiver, (iv) burden OR burdened OR care burden OR caregiver burden OR care-giving burden OR caregiver burden OR caregiver burdens OR burden of caregivers OR stress OR distress OR powerlessness OR burnout OR feelings of burden OR burden of care.

### 2.3. Inclusion and Exclusion Criteria

Inclusion criteria for studies included in the systematic review were as follows: (1) Older adults with chronic diseases living in the community, (2) family caregivers caring for older people, and (3) caregiving burden of family caregivers. Conversely, exclusion criteria for studies were as follows: (1) Participants did not belong to the older adult population residing within the community; (2) participants were older adults diagnosed with dementia; (3) caregivers for the older adults were not family members; (4) the study did not focus on caregiving burden experienced by family caregivers; (5) the language used in the study was neither Korean nor English; (6) the study was a duplicate of previously conducted research; (7) the study pertained to animal subjects; (8) the study did not align with appropriate study criteria (e.g., limited to green or gray literature). The inclusion criteria were delineated based on the PIO framework, wherein “P” denotes the population, “I” signifies the intervention of interest, and “O” represents the outcome, as elucidated in the corresponding abstract [13]. In the initial phase of a systematic review, formulating a research question is paramount, as it marks the commencement of the PIO process [14].

The research inquiry centered on the question, “What are the factors contributing to caregiving burden among family caregivers of older adults with chronic illnesses residing in the local community?”. In this context, “P” denoted “family caregivers providing care to older adults aged 65 and above with chronic illnesses residing in the local community”, “I” represented “factors related to caregiving burden of older adults with chronic illnesses”, and “O” encapsulated “caregiving burden experienced by family caregivers of older adults with chronic illnesses residing in the local community”.

### 2.4. Selection Process and Data Extraction

Data retrieval was conducted over a period of three days, commencing on 16 January 2023. To select studies for inclusion, all retrieved studies were reviewed according to the predefined selection and exclusion criteria, and duplicated studies were removed using a bibliographic management program (EndNote 20). After removing duplicate literature in the first round of selection and exclusion, we excluded literature that was unrelated to the research topic by looking at the title and abstract. If it was difficult to judge whether a study was suitable for selection, we checked the text to make a decision. In the second selection/exclusion process, the full text of the literature selected in the first step was reviewed, and literature suitable for the research topic of this study was selected. Two researchers independently evaluated the literature search and selection processes. In the process of selecting literature, if the opinions of the researchers were not in agreement, consensus was reached through discussion. Data extracted from the literature included the number of participants, caregiving burden instruments, factors related to older people with chronic diseases at home, and factors related to family caregivers.

## 3. Results

### 3.1. Selected Documents

**Figure 1:**
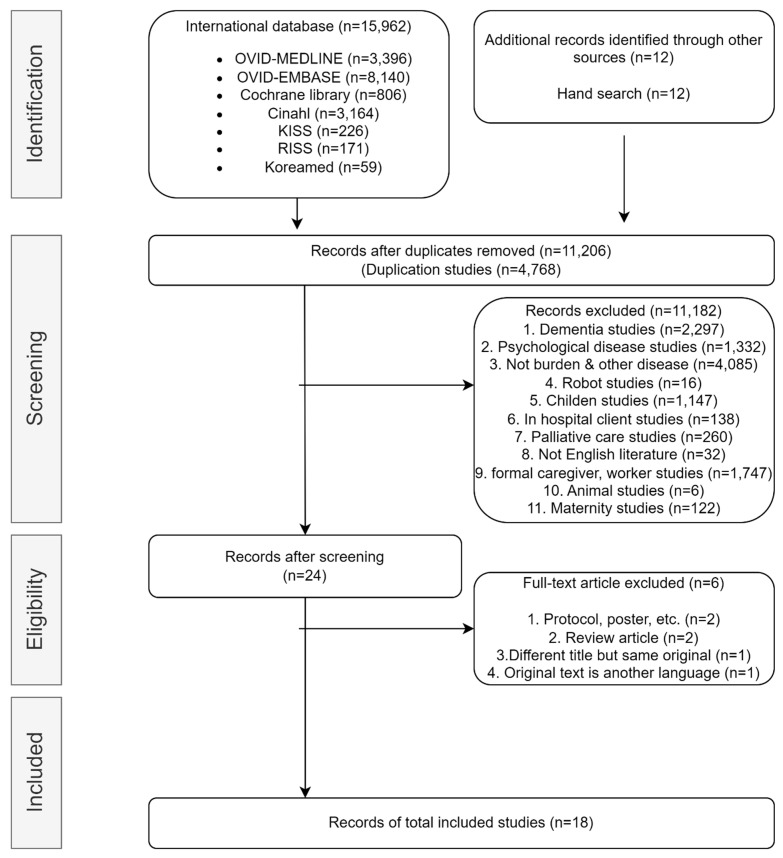
FLOW CHART of selected studies.

Our search retrieved 3396 studies from MEDLINE, 8140 studies from EMBASE, 806 studies from the Cochrane Library, 3164 studies from CINAHL, and 456 studies from domestic electronic databases, for a total of 15,962 studies. Twelve manual searches were included, 4768 duplicate searches were removed, and 11,206 study titles and abstracts were reviewed. Among these studies, those focusing on dementia (2297), psychological diseases (1332), non-burden and other topics (4085), children (1147), and formal caregivers or workers (1747) were excluded. After excluding 11,182 studies unrelated to the research topic, 24 were initially selected. After reviewing the full text of the 24 studies, six were excluded: One study with the same title, one study with the full text in Spanish, two review articles, and two studies with only an abstract. Therefore, 18 studies were selected as eligible for inclusion in the systematic review (Figure 1).

### 3.2. General Characteristics of Selected Studies

The 18 studies reviewed were conducted in the United States (*n* = 2), Singapore (*n* = 2), India (*n* = 2), Taiwan (*n* = 2), Saudi Arabia (*n* = 1), Sweden (*n* = 1), Spain (*n* = 1), Israel (*n* = 1), Indonesia (*n* = 1), Japan (*n* = 1), China (*n* = 1), Canada (*n* = 1), Pakistan (*n* = 1), and South Korea (*n* = 1). The Zarit Burden Inventory was the most commonly used instrument to assess caregiver burden (11 instruments, 61%), followed by the short version of the Zarit Burden Interview (two instruments, 11%), the 24-item Chinese Caregiving Burden Inventory (1 instrument, 5.6%), Japanese version of the Zarit Burden Interview (J-ZBI) (one instrument, 5.6%), Caregiver Burden Scale (one instrument, 5.6%), Burden Scale (one instrument, 5.6%), and Perceived Caregiver Burden Scale (one instrument, 5.6%).

The mean age of participants ranged from 45 to 102 years, with prevalent chronic ailments, including cancer, hypertension, diabetes, musculoskeletal disorders, and stroke. Family caregivers (informal caregivers) ranged in age from 18 to 80 years, and 69.7% were women. In the selected studies, family caregivers were children and spouses of older people, with an average caregiving duration of more than five years (Table 1).

### 3.3. Key Elements Identified through Selected Studies

#### 3.3.1. Family Caregiver Burden Assessment Tool

Seven instruments used to assess caregiving burden of family caregivers with chronic conditions were identified in the selected studies. Caregiving burden was measured using the ZBI in 11 studies and the short version of the ZBI in two studies. In addition, the 24-item Chinese Caregiving Burden Inventory, J-ZBI, Caregiver Burden Scale, Burden Scale, and Perceived Caregiver Burden Scale were each used in one study. The ZBI is one of the most widely used instruments in clinical and research settings for assessing caregiver burden. Developed in 1980, the ZBI serves as a tool to gauge perceived burden experienced by caregivers and encompasses domains such as caregiver health, personal and social life, financial status, emotional well-being, and interpersonal relationships. Initially comprising a 29-item self-report questionnaire, it was condensed into a 22-item version (ZBI-22) in 1985. Further modifications resulted in the creation of a brief 12-item version and a concise 4-item screening version [33]. The 22-item ZBI is the most widely used tool. Caregivers read each interview question, and responses were recorded using a 5-point Likert scale ranging from 0 to 4 points. Scores range from 0 to 21 for little or no burden, 21 to 40 for mild-to-moderate burden, 41 to 60 for moderate-to-severe burden, and 61 to 88 for severe burden. The average range of caregiver burden scores for family caregivers in the selected studies was between mild and moderate (Table 2).

#### 3.3.2. Care Recipient Variables Affecting Caregiving Burden

Table 3 presents statistically significant variables along with caregiving burden. Dependent care burden was found to increase with the age of the care recipient. In addition, decreased ability to perform activities of daily living and increased functional dependency were associated with increased dependent care burden.

Furthermore, caregiving burden is related to type of chronic disease. Cerebrovascular disease, Parkinson’s disease, and urinary incontinence significantly increase caregiving burden. Presence of behavioral problems in the care recipient was a predictor of a higher caregiving burden. In addition, mental issues such as cognitive decline, depression, and sleep disorders in older people were identified as factors that increased caregiver burden (Table 3).

#### 3.3.3. Caregiver Variables That Affect Caregiving Burden

Table 4 summarizes caregiver variables that were significantly related to caregiver burden. Married caregivers, older caregivers, and female caregivers were more burdened. In addition, higher education level was related to a higher burden on caregivers, and children felt less burden of care in relation to older adults. Lower self-reported health status of caregivers was associated with greater caregiving burden, and unemployed caregivers felt more burdened than employed caregivers. Longer caregiving hours were also a factor that increased caregiving burden; caregivers with higher levels of social and family support reported lower caregiving burden (Table 4).

## 4. Discussion

This study aimed to identify characteristics of older adults with chronic diseases living in the community and to analyze factors affecting caregiving burden of family caregivers who support these individuals through a systematic literature review. Furthermore, the study aimed to provide basic data to inform the development of interventions to reduce caregiving burden.

Compared to previous studies on factors that affect caregiving burden for family caregivers of older adults in long-term care, characteristics of older adults included sex, age, educational level, and degree of impairment of physical and daily functioning, which was the most important factor in determining the burden of support. In the case of sex, although the results were not consistent among researchers when caring for an older person with dementia, the burden of supporting an older female person was higher than that of supporting an older male person. In addition, the higher the dependence of older people on others for daily life activities owing to impaired physical function, the higher the burden on caregivers [34,35,36,37,38]. The dependence of older people on others for daily life activities was investigated as an influential factor in caregiving burden, and it was found that the more severe the functional impairment, the greater caregiving burden [39,40]. Mental impairment in older people has also been found to be a strong factor in caregiver burden [34,37,38,41], and cognitive impairment in older people has been reported to be more burdensome than physical impairment [42,43].

With regard to caregiving burden, the main influencing factors were caregiver sex, age, health status, number of secondary caregivers, hours of caregiving per day, education, and monthly income. In terms of sex, female caregivers experience higher levels of burden than male caregivers [37,40,44,45], and caregiving burden increased with age [37,46,47,48,49]. The health of the caregiver is a variable that can be both a result and a cause of caregiving; the poorer the health of the caregiver, the higher the caregiving burden [34,36,37,41,47,49,50,51]. Studies on caregiving hours almost consistently report that longer average daily caregiving hours increase caregiver burden [34,35,48,49,52,53]. The results of this study are consistent with previous findings on factors affecting caregiver burden among family caregivers of older adults receiving long-term care.

The findings also support existing research suggesting that the number of caregiving tasks and the duration of caregiving, both economic and instrumental, have a significant negative impact on family caregivers of older adults with chronic illness. Most of the current literature on caregiver burden focuses on individuals with dementia. Research on caregiving for older adults with dementia supports these findings by showing that the disease status of older adults, age, health status of the caregiver, and education level are significant predictors of caregiver burden.

This study targeted patients with cancer, hypertension, diabetes, musculoskeletal diseases, and stroke, which are relatively common diseases that most people experience during the aging process, rather than older adults with diseases such as dementia, the severity of which is socially recognized and has been primarily covered in existing research. There were no differences in disease type or severity [54]. The inability to perform activities of daily living has been reported to increase the burden on family caregivers of a wide range of patients, including those with dementia and stroke [55]. Patients with geriatric diseases are chronically slow to recover and require long-term care. Children living with older people often take responsibility for caring for these individuals. However, because older adults with geriatric diseases show fewer personality, cognitive, and emotional disorders or problem behaviors than those with dementia, it was expected that severity of disease and period of caregiving would have different effects on caregiver burden.

While societal attention has been focused on family caregivers of older adults with conditions such as dementia, there is a lack of understanding regarding older adults with a wide range of chronic diseases that occur with aging. There is a need for research that differentiates the caregiving experiences of family caregivers according to the type and severity of geriatric illnesses to reduce the burden on family caregivers [54]. Therefore, differentiated types of services should be developed based on these findings.

Considering the factors related to caregiver burden based on previous studies and the results of this study, we found that problematic behaviors and cognitive levels affect caregiver burden. As a cerebrovascular disease, Parkinson’s disease and bed conditions persist, and cognitive impairment and problem behaviors worsen, leading to other symptoms, such as sleep disturbances, that increase caregiver burden. To reduce the burden on family caregivers, various programs are required to improve cognitive function and reduce problematic behaviors, particularly those suitable for older people with chronic diseases living at home.

In addition, it is necessary to make efforts to reduce caregiver dependency by identifying the level of daily life functioning of older people, as assisting with daily life activities creates a significant burden on family caregivers.

Health status was identified as a significant factor that influenced family caregivers. Therefore, a system that simultaneously manages the health status of not only those receiving care but also family caregivers and that provides human resource support, counseling, and education on health problems is required.

In addition, prolonged support delivered by family caregivers can deteriorate their quality of life due to impacts on social activities, leisure activities, and lifestyle changes. Therefore, to reduce caregiving time of family caregivers, it is necessary not only to continue to provide daycare and short-term care facilities for older people with chronic diseases but also to stimulate the participation of family members, reorganize roles, and communicate smoothly. Systematic management is necessary to ensure that self-help groups for family caregivers operate continuously. Therefore, caregivers require substantial and continuous economic support.

### 4.1. Limitations

This study examined family caregivers of older adults with chronic diseases; however, it was not possible to analyze differences in caregiving experiences according to the type and severity of disease. Considering the increase in the number of older adults with various geriatric diseases, verifying the caregiving experiences of family caregivers according to the type and severity of disease is recommended. It is also recommended that future studies compare the care burden factors of institutionalized and homebound older people and develop care programs or protocols for homebound older people.

The limitations of this study include the high heterogeneity of the studies included in the literature review and the difficulty in interpreting the internal stress of family caregivers because of the predominance of quantitative studies. Therefore, future studies should adopt qualitative approaches. Furthermore, the current findings should be interpreted with caution, as continuous and long-term follow-up studies are required to reflect changes in the degree of chronic disease morbidity in the aging population. The absence of an examination of studies that yielded negative results may introduce bias into the research outcomes and limit thorough comprehension and interpretation of the findings. Another limitation pertains to the inability to address publication bias completely within the limitations of this study.

### 4.2. Implications for Practice and Suggestions for Future Research

Considering the diverse factors identified in this study, healthcare professionals should adopt personalized approaches to enhance the well-being of caregivers and older individuals. Community-based support initiatives are crucial, and collaboration between healthcare practitioners and policymakers is recommended to develop programs that offer practical assistance, emotional support, and education to caregivers. Future research should delve deeper into the specific effectiveness of tailored interventions to mitigate the caregiver burden. Comparative studies exploring the outcomes of various community support programs can provide valuable insights into best practices.

## 5. Conclusions

In an upcoming super-aged society, the care of older patients with various chronic diseases living with their families will be the center of discussion; therefore, discussions to support older people with these diseases and their families should continue. It is necessary to understand the impact of each unique disease characteristic on the caregiving burden of family caregivers more comprehensively, to provide specialized support tailored to caregiving burden characteristics, and to provide more realistic medical and non-medical alternatives. As caregiving burden for older patients with chronic diseases is no longer a problem for individuals, healthcare workers should consider the application of integrated nursing interventions that can promote the health of not only patients but also family caregivers simultaneously. In addition, to solve the welfare problems of older people suffering from chronic diseases in the future, efforts in the social community, in which the government, community, and family seek appropriate role sharing, are urgently required.

## Figures and Tables

**Table 1:** Methodological and content-specific variables of the selected studies.

No.	Author/Year	Methodological Variables			Content-Specific Variables					
					Care Recipient Variables		Caregiver Variables			
		Design	Location and Sample Size (*n*)	Burden Measurement	Chronic Illness/Disability State among Surveyed Older Adults	Mean Age	% of Female	Dominant Relationship with Older Adults	Mean Age	Average Care Duration
**1**	Lu et al., 2015 [15]	Cross-sectional	China *n* = 494 older adult–caregiver dyads	24-item Chinese Caregiver Burden Inventory **Approach**: Self-administered questionnaire	Musculoskeletal condition/more than two-thirds of the older adults needed assistance to complete more than two out of ten activities of daily living or equivalent (70.6%)	83.393	51.4%	Children/son-in-law/daughter-in-law: 71.9%	62.645	-
**2**	Kristaningrum et al., 2021 [16]	Cross- sectional	Indonesia *n* = 327 DM patients and their families	ZBI **Approach**: Self-administered questionnaire	Diabetes mellitus	45–65	48.6%	Child: 47.1%	<45	-
**3**	Roudriguez-Gonzalez et al., 2021 [17]	Cross- sectional	Spain *n* = 148 care recipients, 135 caregivers	Zarit Burden Interview **Approach**: Self-administered questionnaire	Chronic illness/ADL mean	≥80	91.1%	-	55–64	≥6 years
**4**	Faison et al., 1999 [18]	Cross- sectional	USA *n* = 88 caregivers of older chronically ill persons	BI(Burden Interview) **Approach**: Self-administered questionnaire	CVD, neurological disorder, psychiatric disease, dementia, Endocrine disorder, etc.	-	77%	Daughters: 54.5%	53.5	>5 years
**5**	Schandl et al., 2022 [19]	Cross- sectional	Sweden *n* = 319 family caregivers	The Caregiver Burden Scale **Approach**: Self-administered questionnaire	Esophageal cancer (adenoCa.)	67	86%	Spouse: 83%	66	-
**6**	Schwartz et al., 2020 [20]	Cross- sectional	USA *n* = 560 informal caregivers	the 22-item Zarit Burden Interview **Approach**: Self-administered questionnaire	Cancer/ADL mean 2.8, IADL mean 5.0	-	77%	Spouse/significant other: 42%	52.6	-
**7**	Hooley et al., 2005 [21]	Cross- sectional	Canada *n* = 50 patients and 50 primary caregivers	ZCB (Zarit Caregiver Burden) **Approach**: Self-administered questionnaire	CHF, HTN, DM, MI	72 ± 11	80%	Spouse: 66%	61 ± 14	-
**8**	Unnikrishnan et al., 2019 [22]	Cross- sectional	India *n* = 205 caregivers of patients with cancer	ZBI (Zarit Burden Interview) **Approach**: Self-administered questionnaire	Cancer	52.6	48%	Children: 38%	42.4	<6 months
**9**	Iecovich, 2008 [23]	Cross- sectional	Israel *n* = 114 primary caregivers	Zarit scale **Approach**: face-to-face interview	Frail older people	79.58	67.5%	Adult child: 70.3%	52.89	5.42 years
**10**	Alshammari et al., 2017 [24]	Cross- sectional	Saudi Arabia *n* = 315 informal caregivers	ZBI-22 **Approach**: Self-administered questionnaire	Chronic problems or disabilities, cognitive impairment, and senility	70–80	52.7%	Children: 69.8%	18~27 (43.8%)	
**11**	Brinda et al., 2014 [25]	Cross- sectional	India *n* = 85 primary caregivers who provided assistance for ADL and accompanied their older care recipients to health facilities	ZBI-22 **Approach**: Self-administered questionnaire	Stroke, dementia, falls, incontinence WHODAS II mean score: 35.2 ± 13.5	74.3 ± 6.7	80%	Co-residence 87.1%	44.2 ± 14.1	38.6 h/week
**12**	Chan et al., 2018 [26]	Cross- sectional	Singapore *n* = 274 patient–caregiver dyads	ZBI-22 **Approach**: Self-administered questionnaire	BI means: 19.48 ± 5.59 Dementia: 50.4% NPI-Q mean: 7.37 ± 6.59	85.29 ± 8	65%	Children: 70.8%	59.1 ± 10.5	88.96 ± 66.13 h/week
**13**	Chen et al., 2015 [27]	Cross- sectional	Taiwan *n* = 108 caregivers of disabled older adults who received home care services with intact cognition	Caregiver Burden Scale **Approach**: Self-administered questionnaire	Total dependence on caregivers: 63.89% ± 25.88 BI score of >/=60:19.86	80.53 ± 7.17	65.74%	Spouse: 81.48%	74.03 ± 6.02	17.50 ± 7.52 h/day
**14**	Choi et al., 2012 [28]	Cross- sectional	South Korea *n* = 267 caregivers	Burden Scale **Approach**: Self-administered questionnaire	Arthritis, hypertension, diabetes mellitus, dementia, stroke, cancer, heart disease	-	62.5%	Children: 47.4%	-	>10 years (40.5%)
**15**	Freeman et al., 2010 [29]	Cross- sectional	Japan *n* = 160 older adults and 84 caregivers	Japan-ZBI (J-ZBI) Burden Index of Care (BIC) **Approach**: Self-administered questionnaire	BI mean scores 80–89 years: 86.7 ± 27.0 90–99 years: 63.5 ± 31.2 100 + years: 44.2 ± 33.9	95.35 ± 7.15	88.09%	Children: 54.4%	63.7 ± 12.75	8.5 ± 7.8 h/day
**16**	Limpawattana et al., 2013 [30]	Cross- sectional	Taiwan *n* = 150 informal caregivers	ZBI-22 **Approach**: Self-administered questionnaire	Hypertension, diabetes, gastric disease, musculoskeletal disease, eye disease, respiration trace disease, and stroke	-	80.67%	Children: 48.67%	51.2 ± 13.7	60 month
**17**	Ong et al., 2018 [31]	Cross- sectional	Singapore *n* = 285 caregivers	ZBI-22 **Approach**: Self-administered questionnaire	Hypertension, hyperlipidemia, diabetes mellitus, mental illnesses including dementia	>60	64.6%	Children: 78.6%	40–65	-
**18**	Sabzwari et al., 2016 [32]	Cross- sectional	Pakistan *n* = 350 caregivers	Perceived Caregiver Burden Scale **Approach**: Self-administered questionnaire	Arthritis, hypertension, diabetes, memory, and agitation requires assistance: 45.7% With an assisted device: 13.7% Bedridden: 3.4%	71.1 ± 10.1	68.9%	Daughter in law 34%	-	47.23 ± 15.5 years

**Table 2:** Assessment categories for caregiver burden.

Name of the Assessment Tool	Mean Range in Selected Studies	Range of Scale	Interpretation of Scores	Summary of Findings
Zarit Burden Inventory Japanese version of Zarit Burden Interview (J-ZBI)	24.09	0–88	0–20: no or little burden 21–40: little to moderate burden 41–60: moderate to severe burden 61–88: severe burden	Little to moderate burden
Short version of the Zarit Burden Interview	29.42	12–60		Moderate level of burden
ZBI’s short version for palliative care	19.6	7–35	17 or more points: “severely” burdened	Higher scores correspond to a greater caregiving burden
24-item Chinese Caregiving Burden Inventory	24.85	0–96	Higher scores indicate a higher degree of burden	High score indicates a high level of burden
Caregiver Burden Scale	25.13	0–60		Resulting scores were used to represent the level of burden. Compared with low caregiver burden, high to moderate burden was associated with reductions in all HRQL aspects.
Caregiver Burden Scale	≥2	1–3.99	1.00–1.99: low burden 2.00–2.99: moderate burden 3.00–3.99: high burden	High to moderate caregiver burden

**Table 3:** Care recipient variables significantly related to caregiver burden.

Personal Variables		
Age	Freeman, 2008 [29]	Care recipient age increases, and it becomes a heavier burden on their caregivers.
Functional ability (ADL/IADL, BI)	Lu, 2015 [15]	Functional health was associated with all five dimensions of burden.
	Roudriguez-Gonzalez, 2021 [17]	Burden severity increases significantly with the level of dependence.
	Faison, 1999 [18]	Increases in ADL were associated with increases in caregiver burden.
	Schwartz, 2020 [20]	ADLs associated with high CGB included feeding and toileting.
	Brinda, 2014 [25]	The dependent older people and the time spent on ADL increased the burden on caregivers.
	Sabzwari, 2016 [32]	The higher the physical and cognitive dependence, the greater the burden on the caregiver.
Chronic illnesses	Roudriguez-Gonzalez, 2021 [17]	Care related to incontinence has the greatest effect on burden.
	Faison, 1999 [18]	A significant correlation was observed between incontinence and caregiver burden.
	Hooley, 2005 [21] Brinda, 2014 [25] Sabzwari, 2016 [32]	Increased caregiver burden is associated with disease burden. CVD, Parkinsonism, higher disability, and urinary incontinence significantly worsened the burden. Stroke was significantly associated with perceived caregiver burden.
Behavioral problem Cognition/Mental disorder	Lu, 2015 [15] Roudriguez-Gonzalez, 2021 [17] Sabzwari, 2016 [32] Lu, 2015 [15] Hooley, 2005 [21] Sabzwari, 2016 [32]	Behavioral problems seemed to be the most demanding stressor of caregiver burden. Burdens are aggravated when the patient has behavioral problems. The behavioral problem of the older people was a predictor of increasing the burden on caregivers. Caregiver’s cognitive status affects different dimensions of caregiver burden. Caregiver burden and patient depression score were significantly correlated. Older people with difficulty sleeping were predictors of a higher caregiver burden.

**Table 4:** Caregiver variables significantly associated with caregiver burden.

Personal Variables		
Age	Schandl, 2022 [19]	Younger family caregivers were more likely to have a higher burden on caregivers.
	Unnikrishnan, 2019 [22]	Caregivers of patients who were of older age had moderate to severe burden.
	Limpawattana, 2013 [30]	The age of caregivers had a positive relationship with ZBI scores.
Sex	Unnikrishnan, 2019 [22]	Female caregivers had moderate to severe burden.
	Freeman, 2008 [29]	Male caregivers experienced lower levels of burden compared to female caregivers.
Marital status	Schwartz, 2020 [20]	Variables associated with high CGBs included married people.
Relation	Faison, 1999 [18]	Sons reported significantly less burden than did either daughters or others.
	Freeman, 2008 [29]	Male caregivers, who are biological children, experienced lower burdens than female caregivers.
Education	Lu, 2015 [15]	The higher the level of education, the higher the level of developmental burden.
	Schwartz, 2020 [20]	High CGBs have been reported at educational levels above 4-year college degrees.
Duration of caregiving	Lu, 2015 [15]	Shorter informal care hours were associated with lower levels of physical burden.
	Brinda, 2014 [25]	Time spent on helping ADL and on supervision increased the caregiver’s burden.
	Limpawattana, 2013 [30]	Duration of care had a positive relationship with ZBI scores.
Health status	Roudriguez-Gonzalez, 2021 [17]	Poor caregiver health also contributes to burden levels.
	Schwartz, 2020 [20]	Self-reported poor health was reported as a high CGB.
	Hooley, 2005 [21]	ZBI scores were associated with an increased number of medications and comorbidities.
	Chen, 2015 [27]	Lower physical health and higher caregiver burden scores.
	Choi, 2010 [28]	There were significant correlations between health status and the burden of the family caregiver.
	Limpawattana, 2013 [30]	Self-reported health status had a positive relationship with ZBI scores.
Employment status	Roudriguez-Gonzalez, 2021 [17]	Not being retired also contributes to burden levels.
	Unnikrishnan, 2019 [22]	Caregivers of patients who were unemployed had moderate to severe burden.
	Freeman, 2008 [29]	Employed caregivers experienced less burden than unemployed caregivers.
Income	Schwartz, 2020 [20]	Higher income levels were reported as higher CGBs.
	Hooley, 2005 [21]	Caregivers with lower income had higher caregiver burden ZCB scores.
	Limpawattana, 2013 [30]	Self-reported income had a negative relationship with ZBI scores.
	Sabzwari, 2016 [32]	Financial impact had a strong correlation with perceived caregiver burden.
Support (family, social)	Iecovich, 2008 [23]	Caregiver burden increased according to the availability of formal community-based services.
	Choi, 2010 [28]	There were significant correlations between family support and caregiving burden.
	Ong, 2018 [31]	Caregivers with a higher level of social support experience a lower level of burden.

## Data Availability

The data presented in this study are available upon request from the corresponding author.
